# Cyclodextrin enhanced the soluble expression of *Bacillus clarkii* γ-CGTase in *Escherichia coli*

**DOI:** 10.1186/s12896-018-0480-8

**Published:** 2018-11-12

**Authors:** Lei Wang, Sheng Chen, Jing Wu

**Affiliations:** 1 State Key Laboratory of Food Science and Technology, Jiangnan University, Wuxi, 214000 China; 2 School of Biotechnology and Key Laboratory of Industrial Biotechnology, Ministry of Education, Jiangnan University, Wuxi, 214000 China

**Keywords:** Cyclodextrin glycosyltransferase, Cyclodextrin, Chemical chaperones, Overexpression, *Escherichia coli*

## Abstract

**Background:**

Cyclodextrin glycosyltransferases (CGTases) catalyze the synthesis of cyclodextrins, which are circular α-(1,4)-linked glucans used in many applications in the industries related to food, pharmaceuticals, cosmetics, chemicals, and agriculture, among others. Economic use of these CGTases, particularly γ-CGTase, requires their efficient production. In this study, the effects of chemical chaperones, temperature and inducers on cell growth and the production of soluble γ-CGTase by *Escherichia coli* were investigated.

**Results:**

The yield of soluble γ-CGTase in shake-flask culture approximately doubled when β-cyclodextrin was added to the culture medium as a chemical chaperone.

When a modified two-stage feeding strategy incorporating 7.5 mM β-cyclodextrin was used in a 3-L fermenter, a dry cell weight of 70.3 g·L^− 1^ was achieved. Using this cultivation approach, the total yield of γ-CGTase activity (50.29 U·mL^− 1^) was 1.71-fold greater than that observed in the absence of β-cyclodextrin (29.33 U·mL^− 1^).

**Conclusions:**

Since β-cyclodextrin is inexpensive and nontoxic to microbes, these results suggest its universal application during recombinant protein production. The higher expression of soluble γ-CGTase in a semi-synthetic medium showed the potential of the proposed process for the economical production of many enzymes on an industrial scale.

**Electronic supplementary material:**

The online version of this article (10.1186/s12896-018-0480-8) contains supplementary material, which is available to authorized users.

## Background

Cyclodextrin glycosyltransferases (EC 2.4.1.19, CGTase) catalyze the transglycosylation (cyclization, disproportionation, coupling) and hydrolysis of linear α-(1,4)-linked glucans [1]. These enzymes can synthesize cyclodextrins from low-cost starch resources by cyclization. Cyclodextrins are circular α-(1,4)-linked glucans with a hydrophilic surface and an internal cavity that make it possible to form inclusion complexes with various hydrophobic guest molecules [2]. Cyclodextrins synthesized using CGTases are normally mixtures containing 6 (α-cyclodextrin), 7 (β-cyclodextrin) or 8 (γ-cyclodextrin) glucose units [1]. Compared with α- and β-cyclodextrin, γ-cyclodextrin exhibits more advantageous cavity size, water solubility and biodegradation [3]; thus, γ-cyclodextrin has found much wider application in industrial settings. However, the market share of γ-cyclodextrin is small because of its low synthesis yield and high price [4]. The production of γ-cyclodextrin is often performed using a γ-CGTase with an alkaline pH optimum. The extremely low cyclization activity of these γ-CGTases [5, 6], compared with those of α- or β-CGTases, is unfavorable for the production of γ-cyclodextrin [7]. Thus, improving the specific cyclization activity and enhancing the expression of γ-CGTases are important issues.

*Escherichia. coli* (*E. coli*) has often been used for the expression of CGTases [8]. However, inclusion bodies generally accumulate in the crowded cytoplasm or periplasmic milieu of *E. coli* during the protein overexpression process [9].Several strategies have been exploited to reduce the formation of inclusion bodies in vivo, including co-expression of chaperone genes, addition of “chemical chaperones”, and lowering the culture temperature and inducer concentration [9, 10]. Chemical chaperones, usually osmolytes, help cells counter unfavorable physiological conditions by stabilizing the native folding state of proteins and destabilizing partially folded states and early aggregates [11]. Cyclodextrins have recently been shown to be efficient chemical chaperones, assisting protein refolding and suppressing the misfolding and aggregation of proteins both in vitro and in vivo [12, 13].

In the present study, cyclodextrin was employed for the first time as a chemical chaperone in the expression of recombinant γ-CGTase in *E. coli*. In addition, an efficient process control strategy was developed to reduce inclusion body formation during γ-CGTase production.

## Results and discussion

### Effect of chemical chaperones on γ-CGTase production by *E. coli*

**Fig. 1:**
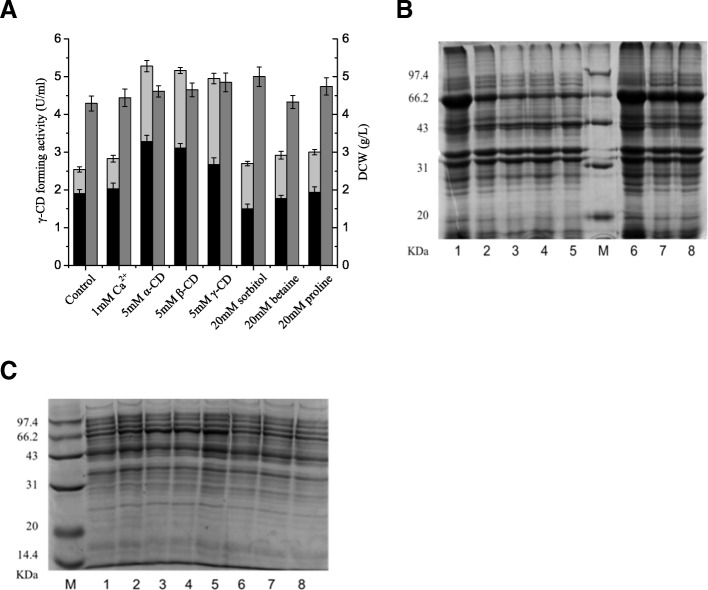
Effect of chemical chaperone candidates on γ-CGTase expression in *E. coli*. **a** Comparison of soluble γ-CGTase activity and cell growth in shake-flask cultures supplemented with different chemical chaperone candidates. Bars represent extracellular γ-CGTase activity (black), soluble periplasmic γ-CGTase activity (light gray), and DCW (gray). CD, cyclodextrin; DCW, dry cell weight. The results were obtained from three independent experiments. **b** and **c** SDS-PAGE analysis of inclusion bodies (**b**) and soluble γ-CGTases (**c**) obtained from shake-flask cultures supplemented with different chemical chaperone candidates. The lanes contain: lane 1, control (TB medium with 0.5%(*w*/*v*) glycine); lane 2, 1 mM Ca^2+^; lane 3, 5 mM α-cyclodextrin; lane 4, 5 mM β-cyclodextrin; lane 5, 5 mM γ-cyclodextrin; lane M, molecular mass markers; lane 6, 20 mM sorbitol; lane 7, 20 mM betaine; and lane 8, 20 mM proline. The band corresponding to γ-CGTase is seen near the 66.2 kDa mass marker

In this set of experiments, we assessed the efficacy of Ca^2+^, proline, betaine, sorbitol, and cyclodextrins as chemical chaperones in shake-flask culture. Ca^2+^ is a ligand of the CGTase molecule and is necessary for maintaining its structure. Proline, betaine and sorbitol have been reported to be beneficial for protein overexpression [11, 14]. No inducer was added to the TB medium, but 0.5% (*w*/*v*) glycine was added to enhance the secretion of γ-CGTase. Thus, the control culture contained 0.5% (w/v) glycine in TB medium, while the test cultures contained, in addition to the glycine, 1 mM Ca^2+^, a 5-mM concentration of α-, β- or γ-cyclodextrin, or a 20-mM concentration of proline, betaine, or sorbitol. Among these chemical chaperone candidates, the cyclodextrins were remarkably effective, compared with the control, in promoting the production of soluble γ-CGTase (Fig. 1a). The total soluble γ-CGTase activity, which is the sum of the extracellular γ-CGTase activity and the γ-CGTase activity extracted from the periplasmic space, obtained in the presence of cyclodextrins was twice that seen with the control (α-cyclodextrin, 2.06-fold; β-cyclodextrin, 2.03-fold; and γ-cyclodextrin, 1.95-fold). The other candidate chemical chaperones elicited increases of less than 20%, compared with the control.

SDS-PAGE analyses revealed that all of the chemical chaperone candidates were somewhat effective in inhibiting the formation of insoluble inclusion bodies (Fig. 1b), but the cyclodextrins were particularly effective. As seen in Fig. 1b, the densities of the CGTase bands (near the 66.2 kDa mass marker) in lanes 3–5 (α-, β-, and γ-cyclodextrin, respectively) are much lighter than that in lane 1 (control) of Fig. 1b. The cyclodextrins were also particularly effective at increasing the recovery of soluble (extracellular plus periplasmic) CGTase. As seen in Fig. 1c, the densities of the CGTase bands (near the 66.2 kDa mass marker) in lanes 3–5 (α-, β-, and γ-cyclodextrin, respectively) are much greater than that in lane 1 (control). The inhibition of inclusion body formation was probably related to the interaction between the cyclodextrins and hydrophobic amino acid residues in CGTase, especially aromatic amino acid residues [13]. This sequestration of hydrophobic side chains shifts the thermodynamic equilibrium toward the unfolded state and away from the formation of aggregates, which is initiated by the hydrophobic amino acids. This interaction is clearly reversible, as there was no notable difference in specific activity between the γ-CGTase expressed in *E. coli* with and without β-cyclodextrin (8.959 versus 8.687 U·mg^− 1^, see Additional file 1: Figure S1 and Additional file 2: Table S1).

### The effect of cyclodextrin concentration on soluble γ-CGTase production by *E. coli*

**Fig. 2:**
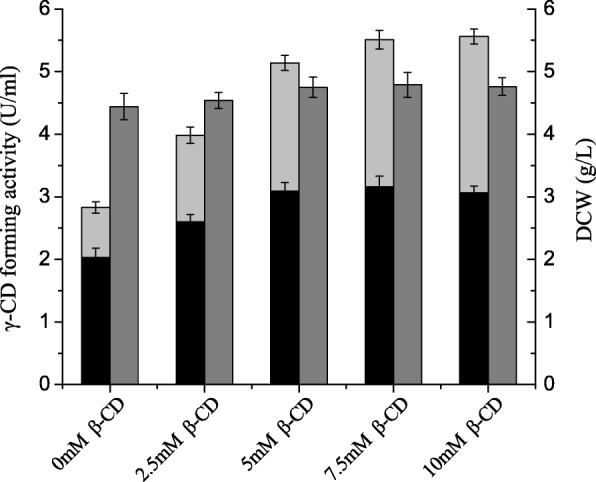
Comparison of soluble γ-CGTase activity and cell growth in shake-flask cultures supplemented with different concentrations of β-cyclodextrin. Bars represent extracellular γ-CGTase activity (black), soluble periplasmic γ-CGTase activity (light gray), and DCW (gray). CD, cyclodextrin; DCW, dry cell weight. The results were obtained from three independent experiments

In these experiments, we assessed the effect of increasing concentrations (0–10 mM) of β-cyclodextrin on the production of soluble γ-CGTase. β-Cyclodextrin was used because it is cheap and readily available, and it effectively promotes the production of soluble γ-CGTase. As shown in Fig. 2, the total soluble γ-CGTase activity increased as the β-cyclodextrin concentration increased until it reached a plateau at a β-cyclodextrin concentration of approximately 7.5 mM. At this point the total soluble γ-CGTase activity had reached 5.51 U·mL^− 1^. In addition, the ratio of extracellular γ-CGTase activity to total γ-CGTase activity decreased from 71.7 to 55.0% when the concentration of β-cyclodextrin increased from 0 to 10 mM.

### The effect of glycine concentration on γ-CGTase production by *E. coli*

We have recently shown that the addition of glycine to the culture medium improves the secretion of recombinant proteins in *E. coli* because it increases the permeability of the *E. coli* cell membrane [15]. Thus, glycine should also influence the diffusion of cyclodextrins across the *E. coli* cell membrane, thereby augmenting their ability to influence protein production.

**Fig. 3:**
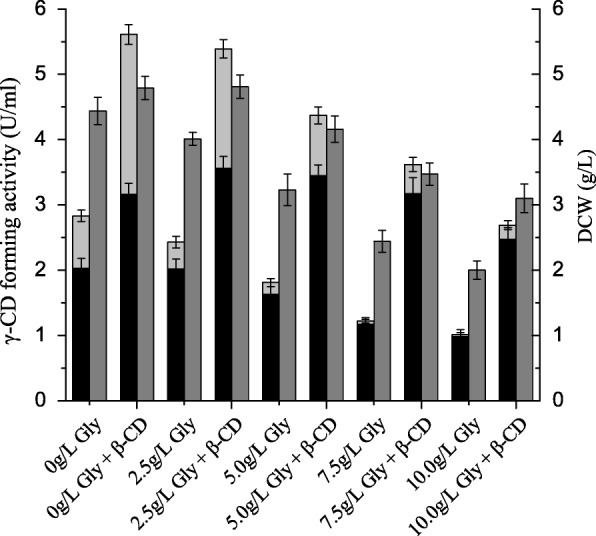
Comparison of soluble γ-CGTase activity and cell growth in shake-flask cultures supplemented with different concentrations of glycine. Bars represent extracellular γ-CGTase activity (black), soluble periplasmic γ-CGTase activity (light gray) and DCW (gray). CD, cyclodextrin; DCW, dry cell weight. The results were obtained from three independent experiments

As shown in Fig. 3, both total soluble γ-CGTase activity and cell growth decreased when the concentration of glycine increased from 0 to 10 g·L^− 1^. However, the ratio of extracellular γ-CGTase activity to the total soluble γ-CGTase activity increased, in both control cultures (from 71.7 to 97.0%) and cultures supplemented with β-cyclodextrin (from 56.3 to 91.8%). More importantly, the effect of β-cyclodextrin on total soluble γ-CGTase activity, as a multiple of the control value, increased from 1.98-fold (5.61 vs 2.83 U·mL^− 1^) to 2.97-fold (3.17 vs 1.17 U·mL^− 1^) as the glycine concentration increased. Thus, the increase in *E. coli* cell membrane permeability mediated by glycine did appear to enhance the diffusion of β-cyclodextrin into these cells, but this effect was second to the detrimental effect of glycine on cell growth and protein production.

### The effect of the induction conditions on γ-CGTase production by *E. coli*

#### Temperature

**Fig. 4:**
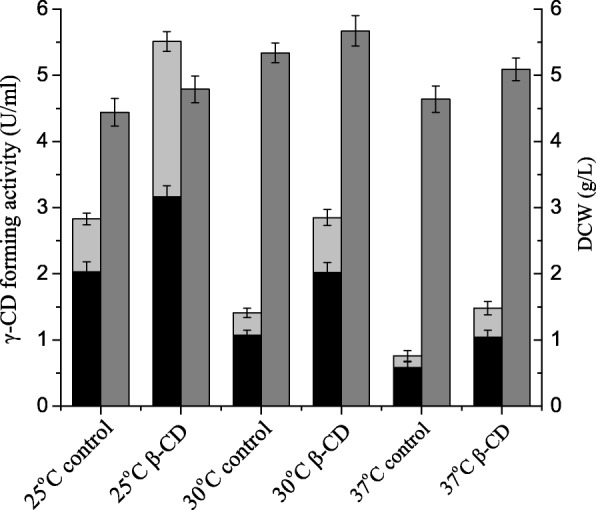
Comparison of soluble γ-CGTase activity and cell growth in shake-flask cultures grown at different temperatures. Bars represent extracellular γ-CGTase activity (black), soluble periplasmic γ-CGTase activity (light gray) and DCW (gray). CD, cyclodextrin; DCW, dry cell weight. The results were obtained from three independent experiments

Induction temperature is an important parameter for recombinant protein production in *E. coli*. In previous studies of the production of recombinant α-CGTase, pullulanase, arginine deiminase and glutamate decarboxylase, we have shown that temperature has important impacts on both cell growth and recombinant enzyme production. In general, induction at lower temperatures increases the solubility of the recombinant proteins by preventing the formation of inclusion bodies. To assess the effect of induction temperature on γ-CGTase production, *E. coli* cells producing γ-CGTase were cultured at 25, 30, or 37 °C. As shown in Fig. 4, soluble γ-CGTase activity decreased as the culture temperature increased. The amount of soluble γ-CGTase produced at 37 °C was less than 30% of that produced at 25 °C for both the control and the culture supplemented with 7.5 mM β-cyclodextrin. However, it should be noted that no matter which temperature was used during the induction, a greater amount of soluble γ-CGTase was produced in the presence of β-cyclodextrin than in its absence (25 °C, 1.98-fold; 30 °C, 2.04-fold; and 37 °C, 2.11-fold). These results demonstrate that adding β-cyclodextrin does not change the effect of induction temperature on the production of soluble γ-CGTase. Rather, β-cyclodextrin addition approximately doubled the production of total soluble γ-CGTase at each temperature. In addition, the ratios of extracellular γ-CGTase activity to total soluble γ-CGTase activity were (25 °C, 71.7%; 30 °C, 75.9%; and 37 °C, 76.3%) in control cultures and (25 °C, 56.3%; 30 °C, 70.9%; and 37 °C, 70.3%) in cultures supplemented with β-cyclodextrin.

#### IPTG and lactose concentration

**Fig. 5:**
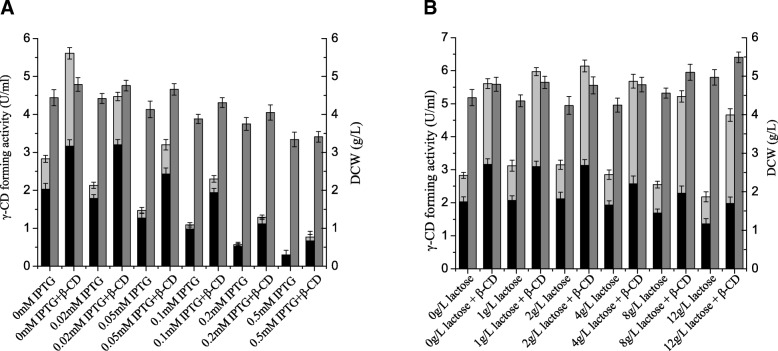
Comparison of γ-CGTase activity and cell growth in shake-flask cultures supplemented with different concentrations of IPTG (**a**) and lactose (**b**), extracellular γ-CGTase activity (black), soluble periplasmic γ-CGTase activity (light gray) and DCW (gray). CD, cyclodextrin; DCW, dry cell weight. The results were obtained from three independent experiments

Since pET-24a (+) is a T7 promoter-based expression system, protein production is usually induced by adding IPTG or lactose to the culture medium. The impacts of IPTG concentration on cell growth and soluble γ-CGTase production were investigated at an induction temperature of 25 °C. As shown in Fig. 5a, the dry cell weight (DCW) decreased with increasing IPTG concentration. Total soluble γ-CGTase production also decreased. When 0.5 mM IPTG was added, total γ-CGTase production in the absence and presence of 7.5 mM β-cyclodextrin dropped to 10.6 and 13.7% of those seen in the absence of IPTG (2.83 and 5.61 U·mL^− 1^), respectively. In addition, the ratio of extracellular γ-CGTase activity to total soluble γ-CGTase activity increased from 71.7 to 100% in control cultures and from 56.3 to 87.0% in cultures supplemented with β-cyclodextrin. However, this trend could not overcome the decrease in total γ-CGTase production, so we conclude from these experiments that induction with IPTG is detrimental to *E. coli* cell growth and not suitable for γ-CGTase production.

Since IPTG, which is a strong, persistent inducer, is not suitable for γ-CGTase production in TB medium, the moderate inducer lactose was assessed as an alternative. As shown in Fig. 5b, increasing lactose concentrations increased the DCW, after a minor decrease at low lactose concentration. In contrast, total soluble γ-CGTase production gradually declined, after a minor increase at low lactose concentration. The greatest total soluble γ-CGTase production was obtained when 2 g·L^− 1^ lactose was added. These results demonstrate that the addition of lactose is not detrimental to *E. coli* cell growth and suggest that lactose may be suitable for the induction of γ-CGTase production during large-scale fermentation in a fermenter. In addition, the ratio of extracellular γ-CGTase activity to total soluble γ-CGTase activity changed from 71.7 to 62.4% in control cultures and from 56.3 to 42.5% in cultures supplemented with β-cyclodextrin.

### γ-CGTase production in a 3-L fermenter using a two-stage, fed-batch strategy with β-cyclodextrin addition

Fed-batch culture processes are often used to achieve high cell concentration and improved productivity while minimizing the problems encountered in high-cell-density culture [16]. We have established a fed-batch fermentation strategy that allows high-cell-density *E. coli* cultivation with high-level recombinant α-CGTase production [17]. In this study, in the pre-induction phase, glycerol feeding was initiated at a flow rate of 6.48 mL·h^− 1^ (a specific feeding rate of 5.40 mL·L^− 1^·h^− 1^) and then the feeding rate (*F*_*t*_) was increased exponentially [18] with a controlled specific growth rate (μ_set_) of 0.20 h^− 1^. Thus, the change in *F*_*t*_ with time can be expressed as ${F}_t=6.48{e}^{0.2\left(t-{t}_F\right)}$ (mL·h^− 1^). When the DCW reached 30 g·L^− 1^, the post-induction phase began and the feeding rate was changed using the gradient-decreasing method [17].

**Fig. 6:**
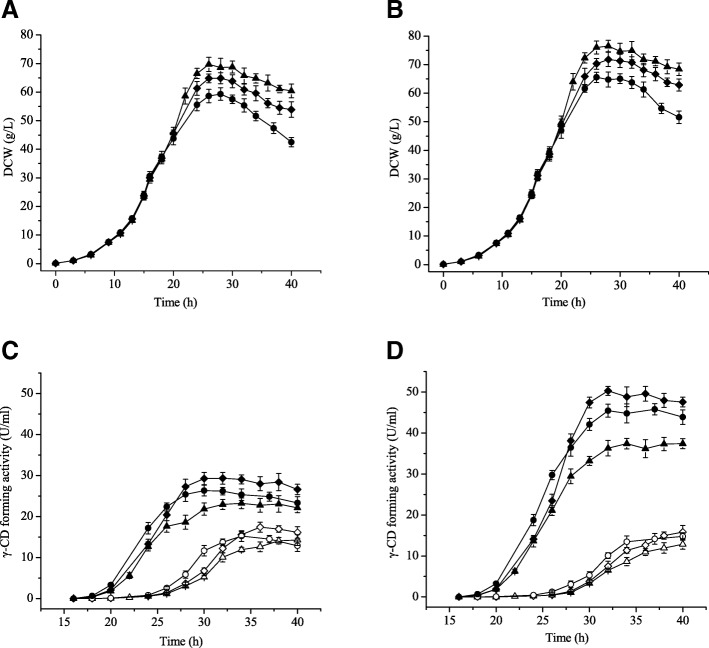
Two-stage glycerol feeding strategy cultivation of *E. coli* and production of soluble recombinant γ-CGTase. **a** The DCW achieved in the absence of cyclodextrin; **b** the DCW achieved in the presence of β-cyclodextrin; **c** the soluble γ-CGTase activity obtained in the absence of β-cyclodextrin; and **d** the soluble γ-CGTase activity achieved in the presence of β-cyclodextrin. Curves represent lactose feeding rates of 0.15 g·L^− 1^·h^− 1^ (triangle), 0.30 g·L^− 1^·h^− 1^ (square) and 0.60 g·L^− 1^·h^− 1^ (circle). The solid plot symbols represent the DCW and total soluble γ-CGTase activity, while the hollow symbols represent the extracellular γ-CGTase activity. CD, cyclodextrin; DCW, dry cell weight. The results were obtained from three independent experiments

The lactose feeding rate were investigated while using a two-stage induction temperature strategy with and without 7.5 mM β-cyclodextrin addition. As shown in Fig. 6, when the induction was performed in the absence of β-cyclodextrin at lactose feeding rates of 0.15, 0.30 and 0.60 g·L^− 1^·h^−^ 1, total soluble γ-CGTase activities of 22.93, 29.33 and 26.18 U·mL^− 1^ were achieved, respectively. However, when 7.5 mM β-cyclodextrin was added, total soluble γ-CGTase activities of 36.30, 50.29 and 45.45 U·mL^− 1^ were achieved. These activities represent 1.58-, 1.71- and 1.74-fold increases over the results obtained in the absence of β-cyclodextrin. The total soluble γ-CGTase activity of the early phase increased with increasing lactose feeding rate. Induction with a lactose feeding rate of 0.30 g·L^− 1^·h^− 1^ gave the highest total soluble γ-CGTase production with or without β-cyclodextrin addition. The greatest total soluble γ-CGTase activity seen in the presence of β-cyclodextrin (50.29 U·mL^− 1^) was the highest production level seen to date. This improvement in γ-CGTase yield has the potential to reduce the cose of γ-cyclodextrin production, which may accelerate the use of γ-cyclodextrin in novel applications. This enthusiasm, however, must be tempered. Although β-cyclodextrin addition increased total soluble γ-CGTase activity, extracellular γ-CGTase activity was not remarkably increased by the adition of β-cyclodextrin. In other words, secretion efficiency appears to be compromised by the addition of β-cyclodextrin, which may due to the protection of *E. coli* cells by β-cyclodextrin, as can be inferred from the increase in DCW.

Since specific productivity (units of enzyme activity per gram of cell weight per hour of culture) is an important parameter in the industrial production of enzymes, the parameters used for recombinant γ-CGTase production in shake flasks and in a bioreactor with fed-batch fermentation are compared in Additional file 3: Table S2. In shake-flask culture, the specific productivity in the presence of β-cyclodextrin increased as the culture temperature decreased from 37 to 25 °C (from 6.6 to 24.0 U·g cell^− 1^·h^− 1^). These levels observed were 1.93- and 1.80-fold greater, respectively, than the levels observed in the absence of β-cyclodextrin. In the case of fed-batch fermentation at 25 °C in a 3-L bioreactor, the specific productivity in the presence of β-cyclodextrin was 1.45-, 1.58-, and 1.55-fold greater than that observed in the absence of β-cyclodextrin at lactose feeding rates of 0.15, 0.30 and 0.60 g·L^− 1^·h^− 1^, respectively.

## Conclusions

**Fig. 7:**
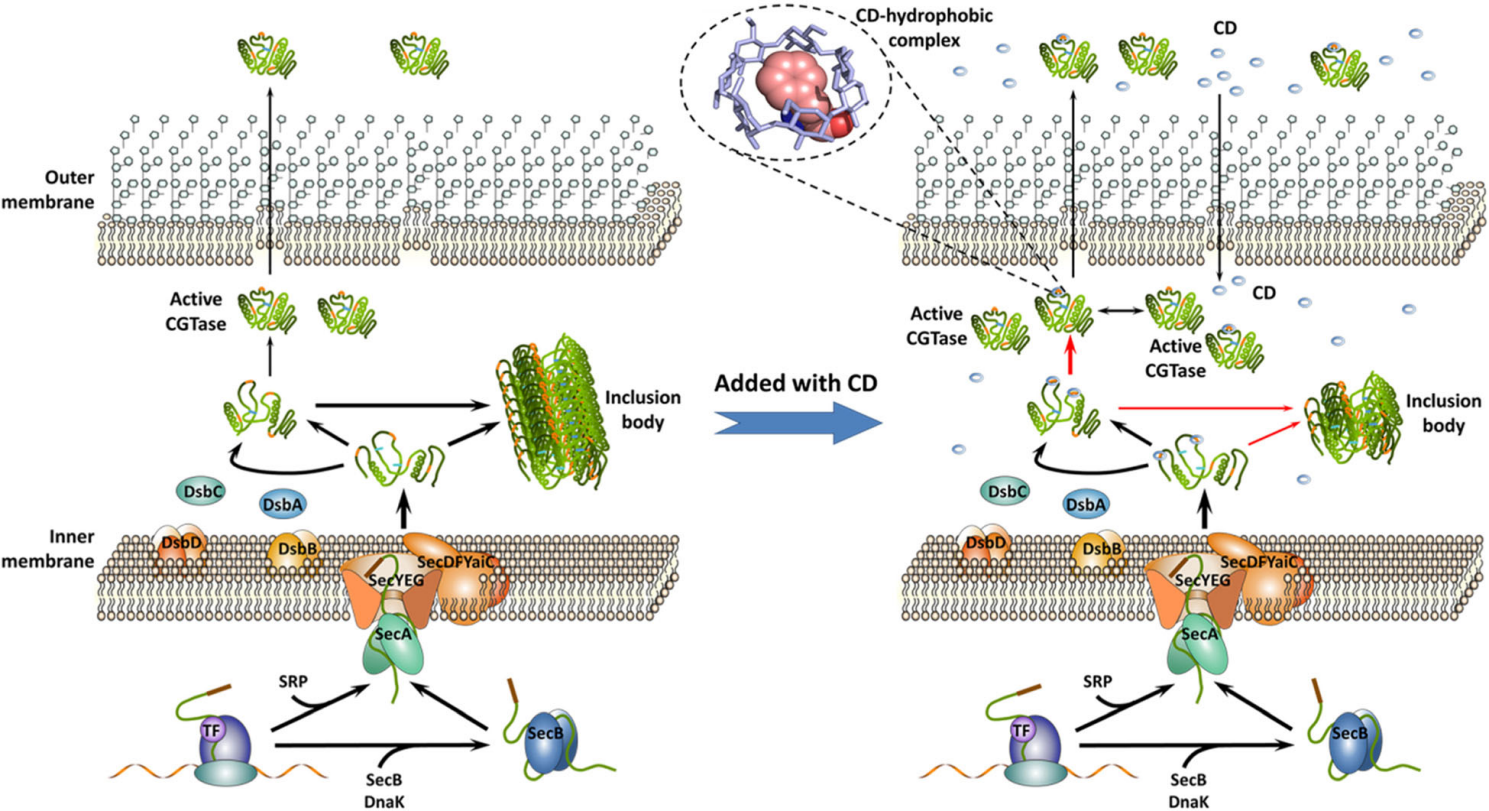
Scheme with simplified protein synthesis and folding pathways comparing CGTase expression by *E. coli* in the absence and presence of cyclodextrin. The unfolded CGTase protein is drawn as a green line, while the signal peptide is drawn as a bold brown line. The orange segments in the green lines represent amino acids with hydrophobic side chains, and the blue rings represent CD molecules. Unfolded CGTase is synthetized by the ribosome complex and transported through the Sec-dependent pathway with the help of cellular proteins SecB, DnaK, SRP and SecA, as well as the membrane protein Sec-complex. In the periplasmic space, unfolded CGTase is either folded into an active protein or misfolded and aggregated into inclusion bodies

In conclusion, we describe the first use of cyclodextrin as a chemical chaperone in the production of recombinant γ-CGTase in *E. coli*. The addition of β-cyclodextrin probably enhancing the soluble expression of γ-CGTase by inhibiting the formation of inclusion bodies (Fig. 1b); a scheme depicting the relevant processes is presented in Fig. 7. The addition of β-cyclodextrin was combined with process optimization strategies to enhance the production of soluble γ-CGTase by *E. coli*. A modified two-stage glycerol feeding strategy in the presence of β-cyclodextrin enhanced the production of soluble *B. clarkii* γ-CGTase in a 3-L fermenter. Through this cultivation approach, the total soluble activity and specific productivity reached 50.29 U·mL^− 1^ (5.61 mg·mL^− 1^) and 22.4 U g^− 1^_cell_ ·h^− 1^, respectively. Since β-cyclodextrin is inexpensive and not toxic to microbes, it has the potential to be universally applied to the economical production of soluble recombinant enymes on large scales.

## Methods

### Bacterial strains, plasmid and materials

The *E. coli* BL21(DE3) expression strain used in this study harbored the recombinant plasmid cgt/pET24a (+), in which a sequence encoding the OmpA signal peptide is fused to the cgt gene encoding γ-CGTase from *Bacillus clarkii* 7364 (Accession No.: BAH14968.1). Yeast extract and tryptone were purchased from Oxoid Co., Ltd., while α-cyclodextrin, β-cyclodextrin, γ-cyclodextrin, soluble starch, bromocresol green, lactose, isopropyl β-D-1-thiogalactopyranoside (IPTG) and all other chemicals and reagents were purchased from Sinopharm Chemical Reagent (Shanghai, China) Co., Ltd.

### Media and feeding solutions

LB medium was used to prepare seed cultures and the TB medium was used in shake-flask cultures, see reference [17]. The medium used for γ-CGTase production in the 3-L fermenter contained (g·L^− 1^): yeast extract 4.0, tryptone 2.0, glycerol 8.0, (NH_4_)_2_HPO_4_ 4.0, KH_2_PO_4_ 10.0, citric acid 1.7, and MgSO_4_·7H_2_O 1.4, as well as 10 mL·L^− 1^ trace metal solution, pH 7.0. The trace metal solution contained (g·L^− 1^): FeSO_4_·7H_2_O 10.0, ZnSO_4_·7H_2_O 5.25, CuSO_4_·5H_2_O 3.0, MnSO_4_·4H_2_O 0.5, Na_2_B_4_O_7_·10H_2_O 0.23, (NH_4_)_6_Mo_7_O_24_ 0.1 and CaCl_2_ 2.0. The feeding solutions were classified into four kinds: (1) a carbon source, which consisted of 500 g·L^− 1^ glycerol plus 30 g·L^− 1^ MgSO_4_·7H_2_O; (2) a nitrogen source, which consisted of ammonia liquor (also served as a base for pH control); (3) an antifoaming agent; and (4) an induction agent, which consisted of 200 g·L^− 1^ lactose. All the media above were supplemented with kanamycin sulfate (physical filter) at a final concentration of 30 mg·L^− 1^.

### Culture conditions

#### Shake-flask

Seed cultures were started by inoculating 50 mL LB medium containing 30 mg·L^− 1^ kanamycin sulfate in a 250-mL flask with 100 μL of a frozen glycerol stock (kept at − 80 °C) of the appropriate organism. The cultures were grown at 37 °C in a rotary shaker at 200 RPM. The overnight seed culture was then diluted (8% *v*/v) into 50 mL of TB medium containing 30 mg·L^− 1^ kanamycin sulfate in a rotary shaker (200 RPM) at 37 °C until an optical density at 600 nm (OD_600_) of 1.0 was reached. IPTG was added to induce expression of the target protein. Incubation was continued for another 48 h at 25 °C unless otherwise stated. At defined time intervals, samples were collected and analyzed for OD_600_, DCW and enzyme activities.

#### Bioreactor

An overnight seed culture was diluted (8% v/v) into a semi-synthetic medium (described in “The effect of cyclodextrin concentration on soluble γ-CGTase production by *E. coli*” section) for fed-batch cultivation. Fed-batch cultivation, which was performed in a 3-L fermenter (BioFlo 115, New Brunswick Scientific Co., Ltd), consisted of three phases that were performed as described by reference [17] with some modifications. The first phase, characterized as a batch phase, had an initial glycerol concentration of 8 g·L^− 1^ and a culture temperature of 37 °C. After inoculation, the dissolved oxygen (DO) value decreased immediately and there was a gradual decrease in pH and glycerol. The end of glycerol consumption was signaled by a sharp increase in both DO and pH value. As the DO and pH value kept growing, the second, exponential feeding (pre-induction) phase of fed cultivation was started. When a DCW of 30 g·L^− 1^ was reached, the inducer was fed at a range from 0.15 to 0.6 g·L^− 1^·h^− 1^and the culture temperature was decreased to 25 °C (unless otherwise stated) for γ-CGTase production. This began the third, post-induction phase. During the whole process, the pH was kept at 7.0 by automatic addition of ammonia solution (25%, *v*/v). Antifoam was added manually when necessary. To maintain the DO level at around 30% of air saturation, the agitation speed was varied from 200 to 900 RPM. The air flow rate was 1.8 L·min^− 1^. Dissolved oxygen concentration, pH, temperature, and impeller speed were recorded using Advanced Fermentation Software (AFS) from New Brunswick Scientific Co. Inc.

#### Determination of biomass

Cell growth was monitored during cultivation as described by Duan [14].

#### Cell fractionation

Cell fractionation was performed as described by Duan [14] with some modifications. The extracellular fraction was obtained by centrifugation of the culture broth at 13,800 *g* for 10 min at 4 °C, and the supernatant was used as the extracellular fraction. The cell pellets arising from the centrifugation were harvested and resuspended in 1 mL of 30 mM Tris–HCl solution (pH 8.0) containing 25% (*w*/*v*) sucrose and 1 mM EDTA. The cell suspension was incubated on ice for 2 h and pelleted by centrifugation at 13,800 *g* for 10 min at 4 °C. The supernatant was collected as the periplasmic fraction. The total soluble γ-CGTase activity was the sum of periplasmic enzyme activity and the extracellular enzyme activity.

#### Assay of γ-CGTase activity

All enzyme assays were performed by incubating 0.1 mL of diluted enzyme (0.35–1.0 U·mL^− 1^) with 2 mL of 2% (w/v) soluble starch in glycine sodium/hydroxide buffer (pH 10) at 50 °C for 10 min. The γ-cyclodextrin-forming activity was determined using the bromocresol green (BCG) method, with some modifications. The reaction described above was terminated by the addition of 3.0 M HCl (0.2 mL), and then 0.5 mL sodium citrate and 0.2 mL 5 mM BCG were added. After the reaction mixture had incubated at 25 °C for 10 min, the amount of γ-cyclodextrin in the mixture was determined spectrophotometrically by measuring the absorbance at 620 nm. One unit of γ-cyclodextrin-forming activity was defined as the amount of enzyme that produced 1 μmol of γ-cyclodextrin per minute.

## Additional files

Additional file 1: **Figure S1.** SDS-PAGE analysis of the purification of soluble, extracellular γ-CGTase expression by *E. coli* with and without β-cyclodextrin added. (DOCX 202 kb)

Additional file 2: **Table S1.** Summary of the purification of soluble extracellular γ-CGTase expressed by *E. coli* with and without added β-cyclodextrin. (DOCX 17 kb)

Additional file 3: **Table S2.** Comparison of parameters for recombinant γ-CGTase production in shake flasks and a 3-L fermenter. (DOCX 18 kb)

## Abbreviations

BCG: Bromocresol green
CD: Cyclodextrin
CGTase: Cyclodextrin glycosyltransferase
DCW: Dry cell weight
DO: Dissolved oxygen
*E. coli*: *Escherichia coli*
EDTA: Ethylenediaminetetraacetic acid
IPTG: Isopropyl β-D-1-thiogalactopyranoside
RPM: Revolutions per minute
SDS-PAGE: Sodium dodecyl sulfate polyacrylamide gel electrophoresis
