# Supplementary material for: Cyclodextrin enhanced the soluble expression of Bacillus clarkii γ-CGTase in Escherichia coli
Source: BMC Biotechnol. 2018 Nov 12;18:72. doi: 10.1186/s12896-018-0480-8 (PMC6233531; doi:10.1186/s12896-018-0480-8)
Supplement: Supplementary file 1 — Figure S1. SDS-PAGE analysis of the purification of soluble, extracellular γ-CGTase expression by E. coli with and without β-cyclodextrin added. (DOCX 202 kb) [file 12896_2018_480_MOESM1_ESM.docx]

**Additional file 1: Figure S1**


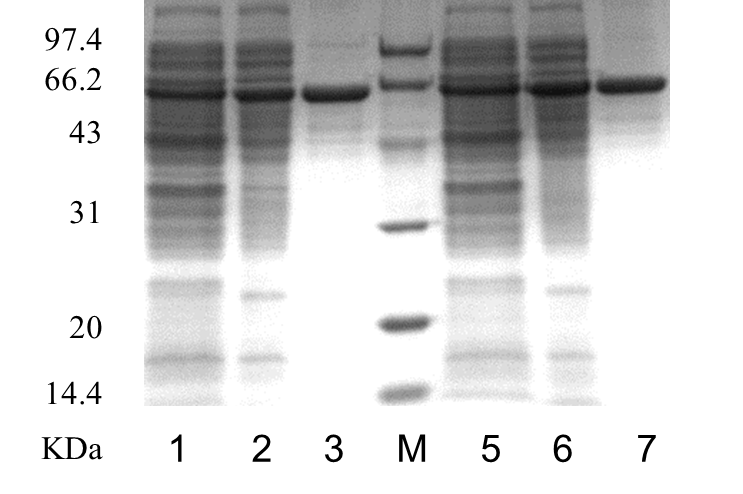


SDS-PAGE analysis of the purification of soluble, extracellular γ-CGTase expression by *E. coli* with and without added β-cyclodextrin. Lanes: 1–3, no β-cyclodextrin added (1, crude enzyme; 2, purified by 25% (NH_4_)_2_SO_4_; 3, purified by MonoQ); M, molecular mass markers. Lanes 4–6, β-cyclodextrin added (4 crude enzyme; 5, purified by 25% (NH_4_)_2_SO_4_; 6, purified by MonoQ).
