# Supplementary material for: Cyclodextrin enhanced the soluble expression of Bacillus clarkii γ-CGTase in Escherichia coli
Source: BMC Biotechnol. 2018 Nov 12;18:72. doi: 10.1186/s12896-018-0480-8 (PMC6233531; doi:10.1186/s12896-018-0480-8)
Supplement: Supplementary file 2 — Table S1. Summary of the purification of soluble extracellular γ-CGTase expressed by E. coli with and without added β-cyclodextrin. (DOCX 17 kb) [file 12896_2018_480_MOESM2_ESM.docx]

**Additional file 2 Table S1** Summary of the purification of soluble extracellular γ-CGTase expressed by *E. coli* with and without added β-cyclodextrin

| Enzyme | Purification step | Total activity（U） | Total protein （mg） | Specific activity (U/mg) | Purification (fold) | Yield |
| --- | --- | --- | --- | --- | --- | --- |
|  |  |  |  |  |  | (%) |
| Control | Crude enzyme | 177 | 345.3 | 0.513 | 1.00 | 100.0 |
|  | 25% (NH_4_)_2_SO_4_ | 139.9 | 92.4 | 1.514 | 2.95 | 79.0 |
|  | MonoQ | 58.81 | 6.77 | 8.687 | 16.93 | 33.2 |
| With added β-cyclodextrin | Crude enzyme | 256.7 | 323.2 | 0.794 | 0.98 | 100.0 |
|  | 25% (NH_4_)_2_SO_4_ | 196.8 | 88.4 | 2.226 | 2.74 | 76.7 |
|  | MonoQ | 83.5 | 9.32 | 8.959 | 11.03 | 32.5 |
