# Supplementary material for: Cyclodextrin enhanced the soluble expression of Bacillus clarkii γ-CGTase in Escherichia coli
Source: BMC Biotechnol. 2018 Nov 12;18:72. doi: 10.1186/s12896-018-0480-8 (PMC6233531; doi:10.1186/s12896-018-0480-8)
Supplement: Supplementary file 3 — Table S2. Comparison of parameters for recombinant γ-CGTase production in shake flasks and a 3-L fermenter. (DOCX 18 kb) [file 12896_2018_480_MOESM3_ESM.docx]

**Additional file 3 Table S2** Comparison of parameters for recombinant γ-CGTase production in shake flasks and a 3-L fermenter.

| Mode of  cultivation | Temperature  (^o^C) | β-cyclodextrin concentration  (mM) | Inducer  (mM or g·L^-1^ or g·L^-1^·h^-1^) | Total γ-CGTase activity (U·mL^-1^) | | Specific productivity  (U g^-1^_cell_ ·h^-1^) |
| --- | --- | --- | --- | --- | --- | --- |
| Shake flask | 37 | 0 | 0 | 0.76±0.17 | 3.4±0.6 | |
|  | 37 | 7.5 | 0 | 1.60±0.21 | 6.6±0.7 | |
|  | 30 | 0 | 0 | 1.41±0.15 | 5.5±0.5 | |
|  | 30 | 7.5 | 0 | 2.87±0.27 | 10.5±1.0 | |
|  | 25 | 0 | 0 | 2.83±0.24 | 13.3±1.4 | |
|  | 25 | 7.5 | 0 | 5.51±0.32 | 24.0±1.8 | |
|  | 25 | 0 | 0.02 | 2.13±0.18 | 10.0±0.8 | |
|  | 25 | 7.5 | 0.02 | 4.47±0.25 | 19.6±1.2 | |
|  | 25 | 0 | 2.0 | 3.15±0.34 | 15.5±1.9 | |
|  | 25 | 7.5 | 2.0 | 6.14±0.36 | 26.9±2.1 | |
| 3-L fermentor | 25 | 0 | 0.15 | 22.93±2.20 | 10.3±0.9 | |
|  | 25 | 7.5 | 0.15 | 36.30±2.41 | 14.9±1.3 | |
|  | 25 | 0 | 0.3 | 29.33±2.53 | 14.1±1.0 | |
|  | 25 | 7.5 | 0.3 | 50.29±2.33 | 22.4±1.2 | |
|  | 25 | 0 | 0.6 | 26.18±1.70 | 14.0±1.1 | |
|  | 25 | 7.5 | 0.6 | 45.45±2.28 | 21.7±1.4 | |
